# IM-NSGAII: A novel approach to boost convergence speed and population diversity in multi-objective optimization

**DOI:** 10.1371/journal.pone.0341439

**Published:** 2026-04-17

**Authors:** Wei Jiang, Zhenhua Xie

**Affiliations:** 1 Department of Electromechanical Control, Guangdong Communication Polytechnic, Guangzhou, Guangdong, China; 2 Department of Information Technology, Hunan Biological and Electromechanical Polytechnic, Changsha, Hunan, China; Huazhong University of Science and Technology, CHINA

## Abstract

Convergence speed and population diversity have long been central concerns in multi-objective evolutionary algorithms. However, the NSGAII algorithm often shows insufficient ability to maintain diversity when facing complex Pareto fronts. To address this issue, an improved NSGAII algorithm (IM-NSGAII) is proposed. First, a population evaluation technique is incorporated after non-dominated sorting to filter and select the best parent population. Second, a sparse population strategy with a high-pressure criterion is employed to guide sparse individuals in local exploration, thereby enhancing population diversity. Finally, a difference operator is introduced to facilitate information exchange among sparse individuals, compensating for the slow convergence speed of the original algorithm. The proposed IM-NSGAII is evaluated against five widely used algorithms on the ZDT, DTLZ, MaF, and WFG benchmark problems. Experimental results demonstrate that IM-NSGAII significantly improves both population diversity and convergence speed.

## 1. Introduction

Over the past two decades, the efficiency of multi-objective evolutionary algorithms (MOEAs) in solving MOPs has attracted considerable attention, leading to the development of various algorithms for handling complex PFs. According to their selection criteria, MOEAs can be broadly classified into three categories: dominance-based MOEAs [1–5], metric-based MOEAs [6–10], and decomposition-based MOEAs [11–15]. The robustness of MOEAs has been demonstrated in [16]. To further accelerate convergence, some studies have incorporated mathematical techniques such as scalarisation methods [17], pairwise optimisation methods [18], and gradient-based methods [19]. Although these methods significantly improve convergence, they often lead to premature convergence and local optima.

Given that mathematical methods can help improve the convergence speed of algorithms, many studies have begun to focus on integrating mathematical techniques with MOEAs to achieve an effective balance between convergence efficiency and population diversity. For instance, [20] introduced a hybrid algorithm using kernel gradients and normal vectors, where the former accelerates convergence and the latter guides the search direction. In [21], gradient information from constrained subproblems was integrated into dominance-based MOEAs to balance convergence and diversity. In [22], the conjugate gradient method was incorporated as a mutation operator to enhance hybrid mutation search, while a feedback mechanism adaptively updated its weight coefficients. However, when addressing MOPs with complex PFs, these methods often underperform, as they prioritise convergence over diversity, leading to underexploration of promising regions and suboptimal solutions.

To enhance population diversity, various approaches have been proposed. For example, Xu et al. [23] introduced a new decision-variable-based indicator to evaluate the optimisation contribution of variables, along with two optimisation schemes. In [24], multiple individual selection criteria were analysed theoretically, and a criterion based on indicator sub-modulus was proposed. In [14], a global decomposition strategy based on infinitesimal analysis was designed to capture PF distribution information for adaptive reference vector adjustment. In [25], crossover operators were designed according to individual distribution states, and reinforcement learning was employed to select the most suitable operator, together with a weight adjustment mechanism to promote diversity. In [26], a clustered-population strategy with independent evolution and multiple selection criteria was introduced to preserve diversity, along with a new metric for balancing convergence and feasibility. Despite these advances, such algorithms increase computational complexity, and their convergence speed is often compromised when improving population diversity.

In summary, we consider whether focusing on the selection mechanism of elite individuals and the optimization of search operators can achieve an effective balance between convergence speed and population diversity without increasing algorithmic complexity. Motivated by this idea, this study proposes an improved NSGAII algorithm (IM-NSGAII). NSGAII is chosen as the baseline algorithm for several reasons. First, it features relatively low computational complexity, making it suitable for large-scale optimization problems. Second, NSGAII does not rely on gradient information, thereby avoiding the common drawback of premature convergence to local optima observed in gradient-based methods. Moreover, NSGAII is one of the most widely used multi-objective evolutionary algorithms in engineering applications, offering high practicality and ease of implementation. Nevertheless, despite its popularity and utility, NSGAII still exhibits limitations, particularly in maintaining population diversity and accelerating convergence, highlighting the need for further improvements. Inspired by this observation, we note that the environmental selection strategy in SPEA2, together with DE-based local search techniques, may provide a promising avenue for enhancing the performance of NSGAII. The main contributions of this study are summarised as follows:

a. To improve population diversity, an individual removal strategy is proposed. Crowding information of sorted individuals is evaluated, and those with low crowding values are eliminated in time.
b. A new high-pressure criterion is designed to filter out the best individuals and guide subsequent optimisation as a refined population.
c. To enhance convergence speed, a local search strategy is incorporated. Specifically, a differential evolution (DE) operator is employed to generate offspring for heuristic exploration of promising populations.
d. The performance of IM-NSGAII is evaluated against NSGAII, its variants, and other state-of-the-art MOEAs on 28 benchmark problems with varying characteristics. Experimental results confirm that IM-NSGAII achieves highly competitive performance.

## 2. Materials and methods

### 2.1. Related knowedge

To provide a clearer understanding of the improvements proposed in this work, Algorithm 1 presents the pseudo-code of the classical NSGAII in detail.

**Algorithm 1 Pseudo-code of NSGAII**

1: **Input:** Population size *n*, dimension size *D*, number of objective functions *M*.

2: **Output:** Final population *P*

3: Initialize population $P=\{p_{1},p_{2},\ldots ,p_{n}\}$, crossover factor *cr*, mutation factor *mu*, distribution parameters $\eta_{1}$, $\eta_{2}$, iteration counter *it*, and maximum iterations max_it.

4: Evaluate fitness values and perform non-dominated sorting.

5: Calculate crowding distances for all individuals.

6: **while** it≤max_it **do**

7:  Perform binary tournament selection to generate parent population *P*.

8:  Generate offspring population $\tilde{P}$ using crossover and mutation.

9:  Merge parent and offspring populations: $P\cup \tilde{P}$.

10:  Evaluate fitness values and update the non-dominated sorting results.

11:  Recalculate crowding distances for all individuals.

12:  Increment iteration counter: *it* = *it* + 1.

13: **end while**

14: **Return** the final population $P=\{p_{1},p_{2},\ldots ,p_{n}\}$.

Population individuals *x*_*i*_ are initialized according to Eq 1:


$$x_{i}=lp+(up-lp)\cdot rand(1,D)$$ (1)

where $lp\in R^{1\times D}$ and $up\in R^{1\times D}$ denote the lower and upper bounds of the decision variables, respectively. The function *rand*(1,*D*) generates a *D*-dimensional decision vector with values uniformly distributed in the interval [0,1].

The fitness value for each individual is calculated as follows: for $\forall x_{i}\in X$, the vector *F*(*x*_*i*_) is obtained using Eq 8.

Furthermore, the crowding distance of each individual $x_{k}\in X$ is calculated according to Eq 2:


$$ods(x_{k})=\sqrt{\sum_{i=1}^{m}{\left(f_{i}(x_{k+1})-f_{i}(x_{k-1})\right)}^{2}}$$ (2)

where *f*_*i*_(*x*_*k*_) is the *i*-th objective function of *F*(*x*), and *x*_*k*+1_ and *x*_*k*−1_ are the neighbouring individuals of *x*_*k*_. This crowding distance is used to maintain the distribution of solutions across the Pareto front.

#### 2.1.1. Population individual removal strategy.

In the classical NSGAII, the effective selection of individuals is often applied only to the last non-dominated front using the crowding distance criterion, in order to truncate the population to size *N*. However, this approach may result in an uneven distribution of solutions along the Pareto front. To overcome this limitation, a refined individual removal strategy is introduced in this work.

First, the crowding distance of all individuals in the population is computed. Then, two individuals with the smallest crowding values, denoted as *x*_*i*_ and *x*_*j*_, are identified. For these two individuals, there always exists another individual *x*_*k*_ that is the closest to them in the objective space. To maintain population diversity, one of the individuals *x*_*i*_ or *x*_*j*_ is removed according to Eq 3.


$$\begin{array}{c} x^{\prime }=\left\{ \begin{array}{cc} x_{i}, & \text{if }s(x_{i},x_{k})\leq s(x_{j},x_{k})\\ x_{j}, & \text{if }s(x_{i},x_{k})>s(x_{j},x_{k}) \end{array}\right. \end{array}$$ (3)

$$s(x_{i},x_{j})=\sqrt{\sum_{k=1}^{m}{\left(f_{k}(x_{i})-f_{k}(x_{j})\right)}^{2}}$$ (4)

where *f*_*k*_(*x*) represents the *k*-th objective function of *F*(*x*), and $x_{i},x_{j}\in X$ are the candidate solutions. This mechanism ensures that individuals located in crowded regions are more likely to be removed, thereby promoting a more uniform distribution of solutions across the Pareto front.

The pseudo-code of the proposed population individual removal strategy is given in Algorithm 2.

**Algorithm 2 Population Individual Selection Strategy**

1: **Input:** Population *P*, population size *N*.

2: **Output:** Refined population *P*.

3: Initialize the population size |P|.

4: **if** |P|≤N **then**

5:  Keep the population unchanged: *P* = *P*.

6: **else**

7:  Calculate fitness values and crowding distances for *P*.

8:  Identify individuals *x*_*i*_, *x*_*j*_ with the lowest crowding distances, and find their nearest neighbour *x*_*k*_.

9:  Remove one individual $x^{\prime }$ according to Eq 3.

10:  Update the population: $P=P⧵\{x^{\prime }\}$.

11:  Decrease the population size: |P|=|P|−1.

12: **end if**

13: **Return** the refined population *P*.

#### 2.1.2. Elite populations.

An elite population is introduced to retain individuals that meet specific selection criteria from both parent and offspring populations during each iteration. Its primary purpose is to identify and preserve the most valuable individuals for exploitation, thereby improving the convergence performance of the algorithm. Experimental results demonstrate that incorporating an elite population can significantly accelerate convergence while maintaining a well-distributed population.

In this study, the criteria for elite selection are defined as follows. First, elite individuals must belong to the first non-dominated front of the population, meaning they are among all non-dominated solutions. Second, the crowding distance of an elite individual must exceed a certain threshold *r*, ensuring that elite individuals are sufficiently isolated from others in the objective space to maintain diversity and prevent premature convergence.

The selection of the threshold *r* is crucial for algorithm performance. If *r* is too small, the elite population may include individuals that are not meaningfully distinct from one another, which increases computational overhead without significantly improving the algorithm’s effectiveness. Conversely, if *r* is too large, the number of elite individuals becomes very limited, reducing the algorithm’s ability to exploit promising regions effectively. To overcome this issue, an adaptive *r*-value strategy is proposed, calculated as:


$$r=mean(\sum_{i=1}^{N}ods(x_{i}))$$ (5)

where *ods*(*x*_*i*_) denotes the crowding distance of individual *x*_*i*_. Using this threshold, the elite population is defined as:


$$Elite=P\cap x_{i}|ods(x_{i})>r$$ (6)

Here, *r* represents the mean of the sum of Euclidean distances between all population individuals and their nearest neighbours. Since the population *X* evolves with each iteration, the value of *r* is dynamically updated, allowing the elite selection process to adapt to the changing distribution of the population. This adaptive mechanism ensures a proper balance between exploitation and exploration, preserving both high convergence speed and population diversity. By maintaining a flexible elite population, the algorithm can effectively focus search efforts on promising regions while still exploring diverse areas of the objective space, thereby improving the overall optimization performance.

#### 2.1.3. Localised search for elite populations.

The differential evolution (DE) algorithm, as a heuristic optimization method, possesses several notable advantages, including strong search capability, a small number of stable parameters, fast convergence, and independence from gradient information. To enhance information exchange between populations and accelerate the convergence of the algorithm, the DE operator is applied to perform heuristic learning on the elite population. In this work, the elite individuals identified in Equation 7 undergo a DE-based recombination combined with polynomial mutation to generate the offspring population *Offspring*. The operation can be expressed as:


$$\begin{array}{c} \begin{array}{cc} Offspring & =Elite+cr\cdot (Elite-P\mathrm{.1})+(1-cr)\cdot (Elite-P\mathrm{.2})\\ Offspring & =Offspring+cr\cdot (Parent\mathrm{.1}-Parent\mathrm{.2}) \end{array} \end{array}$$ (7)

Here, *P*.1 and *P*.2 denote subpopulations consisting of *k* individuals randomly selected from the parent population, and *cr* is the crossover factor. This strategy allows elite individuals to share information with other members of the population while generating new candidate solutions, thus improving the exploration capability of the algorithm and enhancing convergence in regions of the search space with promising solutions.

### 2.2. IM-NSGAII algorithm framework

#### 2.2.1. Problem definition.

In practice, many real-world problems can be formulated as multi-objective optimisation problems (MOPs), such as logistics path optimisation [27], power system optimisation [28], and shop floor scheduling [29]. In general, MOPs are mathematically expressed as:


$$\begin{array}{c} \left\{ \begin{array}{cc} \min & F\left(x\right)={\left(f_{1}\left(x\right),f_{2}\left(x\right),...,f_{m}\left(x\right)\right)}^{T}\\ & x\in \Omega \end{array}\right. \end{array}$$ (8)

where x∈Ω is the decision vector, $\Omega \subseteq {\mathbb{R}}^{n}$ denotes the decision space, and *F*(*x*): $\Omega \to {\mathbb{R}}^{m}$ is the *m*-dimensional vector of objective functions *f*_*i*_(*x*), mapping the decision space to the objective space.

Unlike single-objective optimisation, the conflicting nature of the functions *f*_*i*_(*x*) in MOPs prevents their individual optima from representing the global optimum. For decision variables *x* and *y*, if they satisfy the following conditions:


$$\begin{array}{c} \left\{ \begin{array}{cc} f_{i}\left(x\right)\leq f_{i}\left(y\right), & \forall i\in \left\{1,2,...,m\right\},\\ f_{j}\left(x\right)<f_{j}\left(y\right), & \exists j\in \left\{1,2,...,m\right\}. \end{array}\right. \end{array}$$ (9)

then *x* is said to dominate *y*.

In principle, the optimal solution set of MOPs consists of Pareto-optimal solutions. The collection of all Pareto-optimal solutions is referred to as the Pareto set (PS), and the Pareto front (PF) represents the corresponding set of points in the objective space obtained by mapping the PS through *F*(*x*) [30].

#### 2.2.2. Motivation.

NSGAII is one of the most widely used multi-objective evolutionary algorithms, and its effectiveness has been demonstrated in numerous practical applications. Typical examples include integrated production and inventory scheduling [31], optimization of high-dimensional multi-objective initial cable forces in arch bridges constructed by the cantilevered cast-in-place method [32], and multi-objective path planning for mobile robots [33].

**Fig 1:**
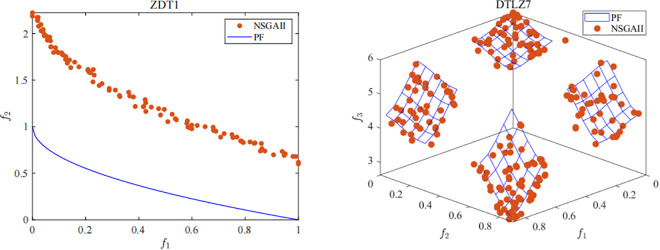
Final solution sets obtained by NSGAII.

Despite its broad applicability, the classical NSGAII algorithm exhibits limitations in maintaining population diversity when addressing complex multi-objective optimization problems. To intuitively illustrate this issue, we analyze the performance of existing algorithms [34–36] on benchmark problems with complex Pareto fronts characterized by non-convexity, discontinuity, and multimodality. Taking the benchmark functions ZDT1 and DTLZ7 as examples, we applied the classical NSGAII algorithm, and the resulting final solution sets are shown in **Fig 1**. The results indicate that the left panel of **Fig 1** demonstrates that, upon reaching the stopping criteria, the algorithm fails to sufficiently approach the true Pareto front, highlighting the urgent need to improve convergence speed. The right panel of **Fig 1** shows that, although the algorithm is able to approximate the true Pareto front, a large number of pseudo-optimal solutions are concentrated in local regions, indicating a tendency to be trapped in local optima. In practical optimization problems, we aim for algorithms that can generate a more uniformly distributed set of solutions to better adapt to environmental changes and decision-making requirements.

These observations motivate the development of the IM-NSGAII algorithm, which is designed to simultaneously enhance population diversity and convergence speed. IM-NSGAII integrates several key mechanisms, including elite population strategies, adaptive differential evolution operators, and crowding-aware individual selection, to generate a more uniformly distributed Pareto front while accelerating convergence. This combination allows the algorithm to explore sparse regions effectively while maintaining robust exploitation of promising areas in the objective space.

#### 2.2.3. General Overview of the IM-NSGAII algorithm.

The proposed IM-NSGAII algorithm extends the classical NSGAII framework by integrating multiple strategies aimed at simultaneously improving convergence speed and population diversity. The key components of the algorithm include a population individual culling strategy, an elite population mechanism, and a differential evolution (DE) operator.

**Algorithm 3 Pseudo-code of IM-NSGAII**

1: **Input:** Population size *N*, dimension *D*, number of objectives *M*

2: **Output:** Final population *P*

3: Initialize population *P*, parameters *cr*, *mu*, $\eta_{1}$, $\eta_{2}$, *it* = 0, *max*_*it*_, $P_{2}=\emptyset$

4: Evaluate fitness and perform non-dominated sorting

5: Compute crowding distances

6: **while** *it* < *max*_*it*_ **do**

7:  Tournament selection to get parent population *P*

8:  Generate offspring *P*_1_ via crossover and mutation

9:  Combine: $P=P\cup P_{1}\cup P_{2}$

10:  Non-dominated sorting; record front numbers

11:  Compute *temp* from nus≤max_nus

12:  **if** ∑(temp)<N **then**

13:   *P*_1_ = *P*(*temp*)

14:  **else**

15:   *P*_3_ from Algorithm 2 and crowding distances

16:  **end if**

17:  Compute adaptive mean *r* (Eq 5) and identify elite *P*_4_ (Eq 7)

18:  Update population: $P=P_{3}\cup P_{4}$

19:  *it* = *it* + 1

20: **end while**

21: **Return** *P*

The population individual culling strategy selectively removes individuals with lower crowding values while retaining those with higher crowding as parents. This approach ensures that the retained individuals are distributed more evenly across the objective space, preventing clustering in dense regions and promoting a uniform spread along the Pareto front. Experimental results indicate that this strategy substantially enhances population diversity and facilitates better coverage of the solution space.

The elite population mechanism identifies and preserves individuals from both parent and offspring populations that meet specific adaptive selection criteria. In particular, elite individuals are drawn from sparsely populated regions, ensuring that promising but underrepresented areas of the Pareto front are actively explored. This mechanism improves the algorithm’s exploratory capabilities and prevents premature convergence to suboptimal regions.

To further accelerate convergence, a differential evolution (DE) operator is applied to the elite population. By generating offspring through DE-based heuristic recombination, elite individuals exchange information with other population members, allowing promising traits to propagate more efficiently. This guided information exchange accelerates convergence while maintaining solution diversity, as it balances exploitation of high-quality solutions with exploration of sparse regions.

The overall workflow of IM-NSGAII is summarized in Algorithm 3. Initially, the population is randomly initialized, and algorithmic parameters—including crossover and mutation factors, crowding coefficients, and maximum iterations—are set. At each generation, the algorithm performs tournament selection to determine parent individuals, generates offspring using simulated binary crossover and polynomial mutation, and combines these with the elite population. Non-dominated sorting is conducted to assign Pareto front numbers, and crowding distances are recalculated to guide selection. An adaptive *r*-value is computed to identify elite individuals dynamically, which are then incorporated into the next generation. The iterative process continues until the maximum number of generations is reached, resulting in a final population that is well-distributed along the Pareto front and provides a rich set of trade-off solutions suitable for practical applications.

Additional Notes:

The adaptive *r*-value ensures that the definition of elite individuals evolves with the population distribution, maintaining a balance between exploration and exploitation.

By integrating DE operators specifically for elite individuals, IM-NSGAII accelerates convergence without sacrificing diversity.

The combination of culling, elite preservation, and DE-guided exploration provides a robust framework that performs effectively across a wide range of benchmark and real-world multi-objective problems.

## 3. Results and discussion

In this paper, IM-NSGAII is compared with the comparison algorithms NSGAII [37], SNSGAII [38], TNSGAII [39], and DRLOSEMCMO [40] on 28 benchmark problems ZDT [41], DTLZ [42], MaF [43], WFG [44], respectively. Among them, NSGAII is the classical algorithm, and the reason for comparing with it is that IM-NSGAII is a variant algorithm of NSGAII, whereas SNSGAII and TNSGAII are variants of NSGAII proposed in 2024 and 2022, respectively. DRLOSEMCMO is a different algorithmic framework from NSGAII, which is also proposed in 2024.

### 3.1. Parameter setting

As for the generation of the offspring solution, NSGAII, TNSGAII and the algorithm IM-NSGAII in this paper use simulated binary crossover and polynomial mutation, where the crossover probability is set, the mutation probability is set to, and the distribution index is set to 1, 1/20, and 20, respectively. Meanwhile, IM-NSGAII introduces the difference operator for the local exploitation of sparse populations. For the algorithms DRLOSEMCMO and SNSGAII, they use their own operators to generate the offspring solutions, where the parameters are defaulted. For problems with two objectives, the population size N was set to 100, and for problems with three objective functions, the population size N was set to 180. *max*_*it*_, the maximum number of iterations, was set to 100 for the ZDT test problem (*max*_*it*_ was 300 for ZDT4). For the DTLZ, MaF, and WFG series of problems *max*_*it*_ is set to 200. specifically, for the three problems DTLZ3, MaF3, and MaF4 *max*_*it*_ is 300. In terms of performance evaluation metrics, IGD [45] and HV [46] are selected to evaluate how well different algorithms perform when facing the 28 benchmark problems. Where HV is used as a (1,1) reference point. The comparative algorithms are tested against IM-NSGAII by running the test problems independently for 30 times on the PlatEMO [47] platform and using the wilcoxon rank sum test with a significance level of 0.05, where + indicates that the algorithm is superior to IM-NSGAII, – indicates that the algorithm is inferior to IM-NSGAII, and = indicates that the algorithm is similar to IM-NSGAII. The CPU model of this experimental equipment is I7-14700KF, MATLAB version is 2021b.

### 3.2. Presentation and analysis of experimental results

Table 1 presents the mean and standard deviation of the IGD values of the five MOEAs over 30 independent runs on the benchmark problems. The results indicate that IM-NSGAII achieves superior performance, with lower IGD values indicating better outcomes.

**Table 1:** IGD values of six algorithms.

Problem	MODE	NSGAII	SNSGAII	TNSGAII	DRLOSEMCMO	IM-NSGAII
ZDT1	6.03e-1 (1.32e-1)-	8.2939e-3 (6.63e-5) -	5.4532e-3 (1.70e-4) -	5.3001e-2 (3.33e-2) -	4.2273e-3 (1.98e-4) -	**4.0813e-3 (1.96e-4)**
ZDT2	1.10e0 (2.50e-1)-	4.3901e-3 (2.02e-4) -	5.4003e-3 (3.12e-4) -	1.2958e-1 (5.82e-2) -	4.0882e-3 (1.19e-4) =	**4.0771e-3 (4.09e-4)**
ZDT3	5.57e-1 (1.15e-1)-	4.9318e-3 (1.39e-4) =	3.3125e-2 (4.58e-2) -	7.5775e-2 (6.58e-2) -	5.6594e-3 (5.31e-4) -	**4.8383e-3 (1.78e-4)**
ZDT4	6.67e0 (2.28e0)-	7.8256e-3 (1.69e-3) -	5.3802e-3 (2.85e-4) -	5.3833e-2 (4.00e-2) -	1.7155e-2 (8.39e-3) -	**4.0544e-3 (2.06e-4)**
ZDT6	1.46e-2 (5.91e-2)-	4.2717e-3 (6.95e-4) -	4.6045e-3 (1.37e-4) -	6.0045e-2 (2.93e-2) -	3.0666e-3 (2.74e-5) =	**3.0580e-3 (1.95e-5)**
DTLZ1	1.43e1 (3.03e0)-	8.3887e-2 (1.17e-1) -	2.0194e-2 (1.18e-3) -	1.5991e-2 (2.16e-4) -	1.3984e-1 (2.52e-1) -	**1.5457e-2 (6.40e-4)**
DTLZ2	7.77e-2 (2.48e-3)-	4.1248e-2 (2.96e-4) =	5.1893e-2 (9.58e-4) -	**3.8842e-2 (6.19e-5)** +	4.4351e-2 (1.04e-3) -	4.1380e-2 (2.00e-4)
DTLZ3	1.31e2 (2.07e1)-	3.4162e-1 (5.42e-1) -	1.6715e + 0 (1.96e + 0) -	9.2915e-2 (6.79e-2) -	2.5165e + 1 (9.81e + 0) -	**6.3885e-2 (1.14e-2)**
DTLZ4	9.80e-2 (1.51e-2)-	4.0933e-2 (4.09e-4) =	5.0023e-2 (9.87e-4) -	3.8819e-2 (1.13e-4) +	4.2367e-2 (5.31e-4) -	**4.0923e-2 (1.13e-3)**
DTLZ5	1.58e-2 (1.75e-3)-	4.3948e-3 (2.25e-3) =	3.2296e-3 (1.79e-4) =	1.4422e-2 (6.71e-3) -	5.1724e-3 (3.56e-4) -	**3.1256e-3 (1.06e-4)**
DTLZ6	2.74e-3 (5.43e-5)	4.0268e-3 (1.64e-3) -	3.2623e-3 (1.22e-4) -	2.5072e-2 (9.54e-4) -	2.4580e-3 (7.32e-6) -	**2.3071e-3 (1.40e-5)**
DTLZ7	6.73e-1 (4.78e-1)-	4.9682e-2 (1.78e-3) -	5.3026e-2 (1.23e-3) -	6.0320e-2 (9.42e-4) -	4.4270e-2 (6.25e-4) -	**4.3591e-2 (3.92e-4)**
IDTLZ1	1.88e1 (8.47e0)-	2.0524e-2 (1.59e-3) -	2.1329e-2 (9.80e-4) -	2.7103e-2 (1.54e-3) -	1.0419e-1 (1.08e-1) -	**1.5860e-2 (2.54e-4)**
MaF1	8.26e-2 (4.13e-3)-	4.7647e-2 (2.41e-4) -	4.3650e-2 (1.76e-3) -	4.9634e-2 (3.81e-4) -	3.7115e-2 (1.30e-3) -	**3.3186e-2 (3.02e-4)**
MaF2	6.69e-2 (2.74e-3)-	4.1943e-2 (6.82e-4) -	3.8319e-2 (1.69e-3) -	2.8902e-2 (1.20e-3) =	3.0717e-2 (1.45e-3) -	**2.8019e-2 (1.20e-3)**
MaF3	2.51e + 4 (6.23e + 3)-	4.3264e-2 (7.39e-2) -	4.7637e-2 (1.40e-2) -	9.2959e-2 (1.20e-2) -	6.9884e + 1 (3.56e-2) -	**3.0260e-2 (1.98ee-2)**
MaF4	5.25e + 2 (3.62e + 1)-	**2.3034e-1 (1.73e-2) +**	2.3905e-1 (2.97e-3) +	3.1611e-1 (7.90e-1) -	7.7371e + 1 (1.47e-2) -	2.6438e-1 (3.01e-3)
MaF5	4.11e-1 (6.10e-2)-	8.1295e-1 (8.81e-1) -	2.2637e-1 (8.55e-3) -	**1.8494e-1 (2.94e-4) =**	1.9755e-1 (5.12e-3) -	1.8673e-1 (6.71e-4)
MaF6	6.78e-2 (1.46e-2)-	3.6794e-3 (7.60e-4) -	2.8929e-3 (4.78e-5) -	1.5745e-2 (3.40e-3) -	4.5199e-3 (1.13e-3) -	**2.6503e-3 (8.86e-5)**
WFG1	1.52e0 (3.97e-2)-	4.5903e-1 (4.82e-2) -	9.8943e-1 (2.74e-1) -	**1.7685e-1 (1.31e-2) +**	8.4391e-1 (6.27e-2) -	2.3219e-1 (2.90e-2)
WFG2	2.94e-1 (1.82e-2)-	1.2610e-1 (1.75e-3) =	4.0466e-1 (1.80e-1) -	1.2625e-1 (1.56e-3) =	1.3944e-1 (4.10e-3) -	**1.2478e-1 (1.69e-3)**
WFG3	3.48e-1 (4.50e-2)-	9.6180e-2 (9.17e-3) =	2.9791e-1 (1.24e-1) -	9.3390e-2 (9.82e-3) =	1.3915e-1 (1.26e-2) -	**9.1068e-2 (5.71e-3)**
WFG4	3.31e-1 (1.08e-2)-	2.2563e-1 (4.32e-3) -	1.9357e-1 (3.88e-3) -	**1.5882e-1 (7.92e-4) +**	1.8789e-1 (6.07e-3) -	1.7593e-1 (2.46e-3)
WFG5	2.21e-1 (1.21e-2)=	2.3709e-1 (1.77e-3) =	2.1161e-1 (9.66e-3) -	**1.7328e-1 (3.51e-4) +**	1.7855e-1 (7.66e-4) +	1.8417e-1 (2.27e-3)
WFG6	3.58e-1 (1.85e-2)-	2.6144e-1 (1.61e-2) -	3.4344e-1 (7.56e-3) -	2.1632e-1 (1.07e-2) =	3.1119e-1 (1.30e-3) -	**2.0991e-1 (9.36e-3)**
WFG7	3.74e-1 (1.26e-2)-	2.1036e-1 (1.73e-3) -	2.2389e-1 (1.26e-2) -	**1.5863e-1 (2.60e-4) +**	1.8228e-1 (4.32e-3) -	1.7298e-1 (3.16e-3)
WFG8	4.72e-1 (1.55e-2)-	2.9016e-1 (1.38e-3) -	3.3115e-1 (1.65e-2) -	**2.5663e-1 (1.69e-3) +**	2.9539e-1 (4.51e-3) -	2.6964e-1 (1.22e-3)
WFG9	3.55e-1 (6.60e-3)-	1.9373e-1 (5.42e-3) -	3.5525e-1 (5.60e-3) -	**1.6344e-1 (2.22e-3) +**	3.1494e-1 (3.17e-3) -	1.7375e-1 (3.62e-3)
+/-/=		1/20/7	1/26/1	8/15/5	1/25/2	

For ZDT1 and ZDT4, IM-NSGAII significantly outperforms the four comparison algorithms. For ZDT2, IM-NSGAII outperforms NSGAII, SNSGAII, and TNSGAII, while DRLOSEMCMO performs similarly to IM-NSGAII. For ZDT3, IM-NSGAII achieves results approximately twice as good as SNSGAII and TNSGAII in terms of magnitude, whereas NSGAII’s performance is comparable to IM-NSGAII. For ZDT6, IM-NSGAII significantly outperforms NSGAII, SNSGAII, and TNSGAII, and is comparable to DRLOSEMCMO.

For the DTLZ series, IM-NSGAII outperforms the comparison algorithms on DTLZ1, DTLZ3–7, and IDTLZ1. For DTLZ2, the algorithm performs slightly worse than TNSGAII, which can be attributed to the high individual selection pressure resulting from a suboptimal value of *r*. Analysis of the Pareto front distribution indicates that the population generated by IM-NSGAII is more uniformly distributed.

For the MaF series, IM-NSGAII achieves the best results on MaF1-3 and MaF6 among all comparison algorithms. TNSGAII produces comparable results only on MaF6. For MaF4, the performance of IM-NSGAII is slightly worse than NSGAII and SNSGAII, which is attributed to the introduction of particle-generated offspring solutions. For MaF5, IM-NSGAII performs similarly to TNSGAII and better than the remaining three algorithms.

For the WFG series, IM-NSGAII achieves the optimal results on WFG2, WFG3, and WFG6. The IGD values of IM-NSGAII on WFG1, WFG3, WFG4, WFG7, WFG8, and WFG9 are lower than those of TNSGAII, with an average difference of approximately 0.133. This difference is partly attributed to variations in the function ranges, which make it challenging for IM-NSGAII to locate the global optima.

Statistical tests indicate that IM-NSGAII significantly outperforms NSGAII, SNSGAII, TNSGAII, and DRLOSEMCMO on 20, 26, 15, and 25 benchmark problems, respectively. Notably, IM-NSGAII converges extremely quickly on the ZDT series, primarily due to its offspring generation mechanism. The performance of IM-NSGAII is similar to that of NSGAII, SNSGAII, TNSGAII, and DRLOSEMCMO on 7, 1, 5, and 2 benchmark problems, respectively.

Table 2 presents the mean and standard deviation of the HV values for the five MOEAs over 30 independent runs. The results show that the probability of IM-NSGAII achieving optimal performance reaches 82%, 75%, 50%, and 79% when compared with NSGAII, SNSGAII, TNSGAII, and DRLOSEMCMO, respectively, across the 28 tested problems. Higher HV values indicate better performance.

**Table 2:** HV values of five algorithms.

Problem	NSGAII	SNSGAII	TNSGAII	DRLOSEMCMO	IM-NSGAII
ZDT1	7.1954e-1 (2.32e-4) =	7.1891e-1 (1.92e-4) -	6.5208e-1 (4.17e-2) -	7.1933e-1 (4.75e-4) =	**7.1977e-1 (5.59e-4)**
ZDT2	4.4349e-1 (3.14e-4) =	4.4364e-1 (2.27e-4) =	3.0693e-1 (4.00e-2) -	**4.4452e-1 (2.51e-4) =**	4.4413e-1 (6.18e-4)
ZDT3	5.9911e-1 (3.27e-4) =	**6.5275e-1 (7.97e-2) =**	5.2349e-1 (7.09e-2) -	5.9839e-1 (5.49e-4) -	5.9954e-1 (3.62e-4)
ZDT4	7.1279e-1 (2.44e-3) -	7.1898e-1 (2.64e-4) =	6.5966e-1 (4.88e-2) -	7.0016e-1 (1.17e-2) -	**7.1952e-1 (4.86e-4)**
ZDT6	3.8588e-1 (1.24e-3) -	3.8738e-1 (1.22e-4) -	3.1211e-1 (3.83e-2) -	**3.8892e-1 (1.79e-5) =**	3.8890e-1 (2.20e-5)
DTLZ1	6.7072e-1 (2.95e-1) -	8.3678e-1 (3.89e-3) -	5.6353e-1 (1.23e-3) -	6.5712e-1 (3.68e-1) -	**8.4556e-1 (3.05e-3)**
DTLZ2	5.3558e-1 (3.90e-4) -	5.5022e-1 (1.62e-3) -	5.6324e-1 (2.42e-4) -	5.5847e-1 (1.57e-3) -	**5.6735e-1 (7.74e-4)**
DTLZ3	3.2859e-1 (2.07e-1) -	9.9923e-2 (2.23e-1) -	**4.8037e-1 (8.25e-2) +**	0.0000e + 0 (0.00e + 0) -	3.5985e-1 (1.75e-1)
DTLZ4	5.3595e-1 (1.13e-3) -	5.5471e-1 (1.02e-3) -	**5.6739e-1 (3.84e-4) =**	5.6292e-1 (1.95e-3) -	5.6556e-1 (2.06e-3)
DTLZ5	1.7419e-1 (2.02e-3) -	**2.0068e-1 (8.78e-5) =**	1.9427e-1 (4.69e-3) -	1.9830e-1 (4.29e-4) -	2.0028e-1 (1.38e-4)
DTLZ6	1.9815e-1 (1.17e-3) -	2.0088e-1 (5.48e-5) -	1.8749e-1 (1.94e-4) -	**2.0119e-1 (1.64e-5) =**	2.0114e-1 (2.22e-5)
DTLZ7	2.7302e-1 (2.46e-3) -	2.8016e-1 (9.68e-4) =	2.7903e-1 (7.78e-4) -	**2.8161e-1 (4.04e-4) =**	2.8181e-1 (4.83e-4)
IDTLZ1	2.1807e-1 (5.42e-3) -	2.1587e-1 (4.67e-3) -	2.0940e-1 (6.16e-3) -	1.2187e-1 (9.80e-2) -	**2.2800e-1 (1.09e-3)**
MaF1	2.1763e-1 (3.68e-4) -	2.1684e-1 (1.12e-3) -	2.1624e-1 (4.98e-4) -	2.1894e-1 (1.86e-3) -	**2.2324e-1 (7.43e-4)**
MaF2	**2.4579e-1 (7.89e-4) +**	2.4049e-1 (1.70e-3) =	2.4473e-1 (1.82e-3) +	2.3787e-1 (1.65e-3) -	2.4184e-1 (7.42e-4)
MaF3	0.0000e + 0 (0.00e + 0) -	**5.2135e-1 (4.79e-1) =**	3.4923e-1 (4.81e-1) =	0.0000e + 0 (0.00e + 0) -	1.8781e-1 (4.20e-1)
MaF4	5.0143e-2 (4.36e-2) -	1.4749e-1 (2.20e-1) -	3.8908e-1 (1.90e-1) +	0.0000e + 0 (0.00e + 0) -	**5.1379e-1 (1.59e-1)**
MaF5	4.7741e-1 (1.18e-1) =	5.5256e-1 (2.92e-3) -	**5.7121e-1 (2.67e-4) +**	5.5494e-1 (3.75e-3) -	5.6522e-1 (1.37e-3)
MaF6	1.9983e-1 (5.34e-4) -	2.0086e-1 (3.74e-5) -	1.9257e-1 (3.17e-3) -	1.9875e-1 (1.03e-3) -	**2.0091e-1 (4.13e-5)**
WFG1	7.3872e-1 (2.60e-2) -	5.1921e-1 (1.37e-1) -	**9.1831e-1 (1.17e-2) +**	5.4640e-1 (3.16e-2) -	8.7563e-1 (1.44e-2)
WFG2	9.2277e-1 (1.69e-3) -	7.6910e-1 (7.72e-2) -	9.2641e-1 (2.03e-3) =	9.1480e-1 (3.39e-3) -	**9.2879e-1 (8.21e-4)**
WFG3	3.8193e-1 (4.76e-3) -	2.9720e-1 (5.56e-2) -	3.7951e-1 (4.12e-3) =	3.6091e-1 (7.07e-3) -	**3.8523e-1 (3.45e-3)**
WFG4	5.3578e-1 (3.51e-3) -	5.4101e-1 (3.80e-3) -	**5.6282e-1 (8.62e-4) +**	5.2857e-1 (5.80e-3) -	5.4234e-1 (3.23e-3)
WFG5	5.0413e-1 (5.10e-3) -	5.1437e-1 (3.28e-3) -	**5.2947e-1 (4.70e-4) =**	5.1465e-1 (8.70e-4) =	5.2497e-1 (3.96e-3)
WFG6	4.8695e-1 (1.23e-2) -	4.1339e-1 (2.39e-3) -	5.1776e-1 (1.01e-2) -	4.2315e-1 (1.32e-3) -	**5.2100e-1 (8.23e-3)**
WFG7	4.2897e-1 (2.96e-3) -	5.1024e-1 (1.82e-2) -	**5.6529e-1 (9.26e-4)** +	5.3665e-1 (3.74e-3) -	5.4673e-1 (1.67e-3)
WFG8	4.3474e-1 (2.56e-3) -	4.3574e-1 (1.56e-2) -	**4.7236e-1 (7.22e-4)** +	4.4934e-1 (4.72e-3) -	4.6611e-1 (9.58e-4)
WFG9	4.9336e-1 (6.27e-3) -	3.9977e-1 (3.73e-3) -	**5.4163e-1 (2.59e-3)** +	4.1403e-1 (1.66e-3) -	5.2408e-1 (3.55e-3)
+/-/=	1/23/4	0/21/7	9/14/5	0/22/6	

**Fig 2:**
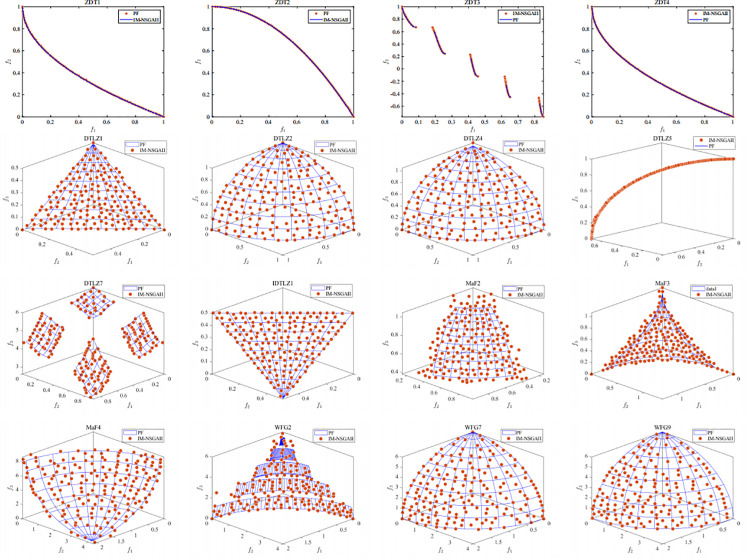
The final solution obtained by IM-NSGAII on the benchmark function.

**Fig 2** illustrates the performance of IM-NSGAII in reaching the optimal solution within the maximum number of iterations. It clearly demonstrates that IM-NSGAII effectively balances convergence speed and population diversity compared to NSGAII.

**Fig 3:**
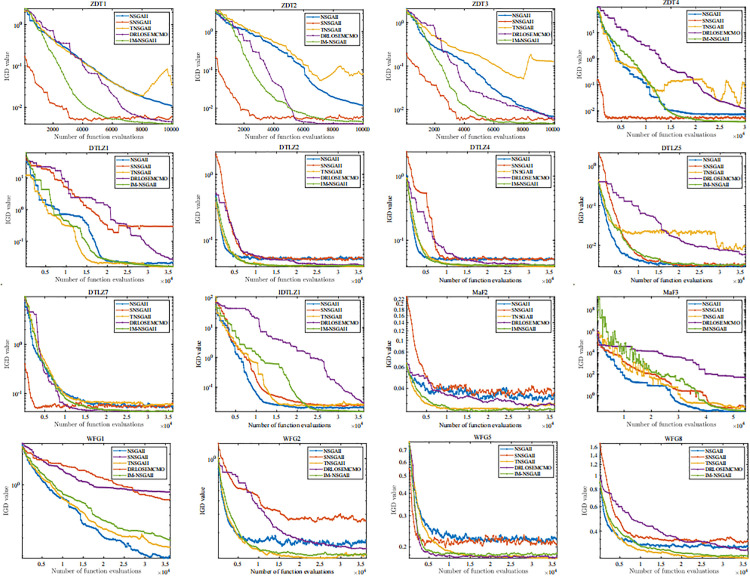
The variation of IGD values with the number of function evaluations on different benchmark functions.

**Fig 3** shows the trend of IGD values for the five algorithms as the number of iterations increases. It can be observed that IM-NSGAII is an improved variant of NSGAII. Specifically, IM-NSGAII achieves the lowest IGD values on ZDT1, ZDT3, ZDT4, DTLZ1, DTLZ2, DTLZ4, DTLZ5, and IDTLZ1. Except for TNSGAII, our proposed algorithm converges the fastest. However, the IGD values of TNSGAII are not as low, indicating that its population diversity is inferior to that of IM-NSGAII.

### 3.3. Ablation study

To comprehensively verify the effectiveness of the three key strategies proposed in the algorithm-namely, the individual elimination mechanism, the elite-population-based adaptive strategy, and the DE-based local search operator-an ablation study was conducted. In particular, we selected two representative benchmark problems with discontinuous and complex Pareto front (PF) characteristics, i.e., ZDT3 and DTLZ7.

Specifically, ZDT3 was employed to evaluate the effectiveness of the DE-based local search operator, while DTLZ7 was used to examine the influence of the individual elimination and elite-population-based adaptive mechanisms on the overall performance of the algorithm. The maximum number of function evaluations for ZDT3 was set to 10,000 with a population size of 100, whereas for DTLZ7, the maximum number of evaluations was set to 42,000 with a population size of 210. During the experiments, a controlled variable approach was adopted to ensure fair comparisons, in which only one strategy was modified at a time while keeping all other parameters identical to the baseline configuration. This setup allows us to clearly isolate and analyze the contribution of each strategy to the algorithm’s convergence ability and population diversity.

As shown in Table 3, the proposed IM-NSGAII (Exp1) achieves the best IGD values on both benchmark problems, demonstrating superior convergence and diversity performance compared to its variants. Specifically, in the ZDT3 problem, IM-NSGAII attains the smallest IGD value of 7.03 × 10^−3^, significantly outperforming the other configurations. When the DE-based local search operator is replaced by the standard GA operator (Exp2), the IGD value increases to 1.04 × 10^−2^, indicating that the DE strategy provides stronger local exploitation ability and facilitates faster convergence toward the Pareto front.

**Table 3:** IGD Values Under Different Strategies.

Problems	Exp1	Exp2	Exp3	Exp4
ZDT3	7.03 × 10^−3^	1.04 × 10^−2^	1.48 × 10^−2^	2.57 × 10^−1^
DTLZ7	4.05 × 10^−2^	4.09 × 10^−2^	5.02 × 10^−2^	7.35 × 10^−2^

Furthermore, when the elite-population-based adaptive mechanism is removed (Exp3), the IGD further deteriorates to 1.48 × 10^−2^, suggesting that the adaptive adjustment of search parameters based on elite individuals effectively enhances the balance between exploration and exploitation. The worst performance is observed in Exp4, where both the crowding-based elimination and elite-adaptive mechanisms are removed, resulting in a substantial degradation of IGD to 2.57 × 10^−1^.

A similar trend can be observed in the DTLZ7 problem. The proposed IM-NSGAII again achieves the smallest IGD value (4.05 × 10^−2^), confirming its robustness and adaptability across different problem landscapes. Overall, these results clearly demonstrate that each designed strategy—particularly the DE-based local search operator and the elite-population-based adaptive mechanism—plays a crucial role in improving the convergence accuracy and maintaining population diversity of the IM-NSGAII algorithm.

### 3.4. Comparison with MODE

To further clarify the role of the Differential Evolution (DE) operator as a local search mechanism, we constructed a comparative variant of the classical NSGAII algorithm by replacing its original Genetic Algorithm (GA) operators (i.e., crossover and mutation) with DE operators, referred to as MODE. MODE was tested on the aforementioned 28 benchmark problems, with the Inverted Generational Distance (IGD) adopted as the primary performance metric. The experimental results, summarized in Table 1, clearly illustrate the search efficiency of MODE.

Across all 28 test problems, IM-NSGAII outperforms MODE on nearly every benchmark, achieving up to two to four orders of magnitude improvement in IGD. The algorithm shows particular strength on DTLZ and MaF problems, indicating its ability to adapt to complex Pareto front structures and maintain the balance between convergence and diversity. These results confirm that the proposed improved mechanisms-such as adaptive mutation, elite learning, and improved crowding distance-enable IM-NSGAII to achieve both higher convergence precision and stronger robustness compared with traditional MODE.

### 3.5. Engineering optimization case

To evaluate the potential extensibility of the proposed IM-NSGAII algorithm, it is applied to two complex multi-objective optimization problems with practical engineering backgrounds. The first problem is an unconstrained multi-objective optimization of a four-bar planar truss, aiming to simultaneously minimize the mass and flexibility of the structure. The second problem is a constrained multi-objective optimization of a two-bar planar truss, where structural performance metrics are optimized under safety constraints.

For both problems, the algorithm is configured with a population size of 100 and a maximum of 50,000 function evaluations. To comprehensively assess the algorithm’s performance, the Hypervolume metric is adopted as the evaluation criterion. The reference points are set as (3.0485281 × 10^3^, 4.0000000 × 10^−2^) and (1.8704862 × 10^2^, 6.7710178 × 10^−5^). The experimental results indicate that IM-NSGAII can effectively maintain solution diversity and approximate the Pareto front when solving complex engineering problems. Furthermore, compared with the benchmark algorithms used above, IM-NSGAII demonstrates significant advantages in both optimization performance and convergence, thereby further validating its potential for broader applications in engineering optimization. The mathematical formulations of the two problems are as follows.

#### 3.5.1. Four-Bar Planar Truss.


$$\begin{array}{cc} & \min f_{1}(x)=L\left(2x_{1}+\sqrt{2}x_{2}+\sqrt{2}x_{3}+x_{4}\right),\\ & \min f_{2}(x)=\frac{FL}{E}\left(\frac{2}{x_{1}}+\frac{2\sqrt{2}}{x_{2}}-\frac{2\sqrt{2}}{x_{3}}+\frac{2}{x_{4}}\right),\\ & \text{s.t.}\;\left\{ \begin{array}{c} \frac{F}{\sigma }\leq x_{1}\leq 3\frac{F}{\sigma },\\ \sqrt{2}\frac{F}{\sigma }\leq x_{2}\leq 3\frac{F}{\sigma },\\ \sqrt{2}\frac{F}{\sigma }\leq x_{3}\leq 3\frac{F}{\sigma },\\ \frac{F}{\sigma }\leq x_{4}\leq 3\frac{F}{\sigma }. \end{array}\right. \end{array}$$ (10)

where $F=10\;\text{kN},E=2\times 10^{5}\;{\text{kN/cm}}^{2},L=200\;\text{cm},\sigma =10\;{\text{kN/cm}}^{2}$.

#### 3.5.2. Two-bar planar truss.


$$\begin{array}{cc} & \min f_{1}(x)=2\rho hx_{2}\sqrt{1+x_{1}^{2}},\\ & \min f_{2}(x)=\frac{\rho h(1+x_{1}^{2}{)}^{1.5}\sqrt{1+x_{1}^{4}}}{2\sqrt{2}Ex_{1}^{2}x_{2}},\\ & \text{s.t.}\;\left\{ \begin{array}{c} g_{1}(x)=\frac{P(1+x_{1})\sqrt{1+x_{1}^{2}}}{2\sqrt{2}x_{1}x_{2}}-\sigma_{0}\leq 0,\\ g_{2}(x)=\frac{P(-x_{1}+1)\sqrt{1+x_{1}^{2}}}{2\sqrt{2}x_{1}x_{2}}-\sigma_{0}\leq 0,\\ 0.1\leq x_{1}\leq 2,\\ 0.5\leq x_{2}\leq 2.5. \end{array}\right. \end{array}$$ (11)

where $\rho =0.283\;{\text{lb/in}}^{3},h=100\;\text{in},P=104\;\text{lb},E=3\times 10^{7}\;{\text{lb/in}}^{2},\sigma_{0}=2\times 10^{4}\;{\text{lb/in}}^{2}$.

**Fig 4:**
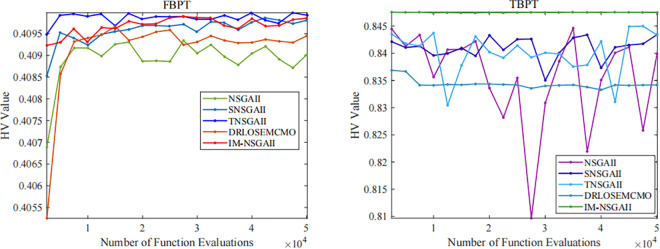
Optimization results of IM-NSGAII in engineering optimization examples.

As shown in **Fig 4**, the proposed IM-NSGAII algorithm demonstrates good optimization performance for both the four-bar planar truss and the two-bar planar truss problems. Specifically, for the four-bar planar truss problem, although the performance metrics of IM-NSGAII are slightly lower than those of TNSGAII overall, the results of both algorithms are nearly identical as the algorithm approaches termination. Compared with the other three benchmark algorithms, IM-NSGAII and TNSGAII exhibit clear advantages in terms of *stability* and the Hypervolume (HV) metric. The observed fluctuations in the performance metrics of IM-NSGAII are mainly attributed to the differential operator and the individual elitism strategy. As a heuristic search mechanism, the differential operator consistently exploits the information of current solutions to explore potential optimal solutions, which explains the overall upward trend of the performance metrics observed in the figure.

For the two-bar planar truss problem, IM-NSGAII achieves the best results. Although the overall improvement in performance metrics is relatively modest, IM-NSGAII demonstrates superior *stability* compared with the benchmark algorithms. Its HV values remain nearly steadily increasing, indicating that the algorithm is capable of maintaining both convergence and solution diversity effectively. These results further demonstrate that IM-NSGAII possesses strong adaptability and robustness when solving different types of engineering optimization problems.

## 4. Conclusions

In this paper, we propose an improved multi-objective evolutionary algorithm (IM-NSGAII) for NSGAII, which can better balance the relationship between convergence speed and population diversity. IM-NSGAII solves the problem of uneven population distribution of the classical algorithm NSGAII by introducing a population individual removal strategy, which tries to retain the optimal solution in each iteration as the parent solution by increasing the selection pressure of population individuals. Uniformity problem. Secondly, the concept and conditions of elite population are customised and the GA operator is used to generate offspring populations to exploit possible unexplored regions during the iteration process. Finally, the DE individual generation method is successfully introduced to replace the simulated binary crossover and polynomial variate generation methods in the classical NSGAII to further improve the convergence speed of the algorithm. The experimental results show that IM-NSGAII effectively improves the convergence speed and population diversity, and is a successful improvement of the algorithm NSGAII.

## Supporting information

S1 Dataset Figures data (Excel file) used in the analyses. (RAR)
